# Linac photon beam fine-tuning in PRIMO using the gamma-index analysis toolkit

**DOI:** 10.1186/s13014-019-1455-1

**Published:** 2020-01-06

**Authors:** Angelina M. Bacala

**Affiliations:** 1 Department of Physics, Mindanao State University-Iligan Institute of Technology, 9200 Iligan City, Philippines; 2 Mindanao Radiation Physics Center, Premier Research Institute of Science & Mathematics, Mindanao State University-Iligan Institute of Technology, 9200 Iligan City, Philippines

**Keywords:** Monte Carlo linac simulation, PRIMO, Linac beam fine-tuning, Gamma-index analysis acceptance criteria, Amazon.com elastic compute cloud

## Abstract

**Background:**

In Monte Carlo simulations, the fine-tuning of linac beam parameters to produce a good match between simulated and measured dose profiles is a lengthy, time-consuming and resource-intensive process. The objective of this study is to utilize the results of the gamma-index analysis toolkit embedded inside the windows-based PRIMO software package to yield a truncated linac photon beam fine-tuning process.

**Methods:**

Using PRIMO version 0.1.5.1307, a Varian Clinac 2100 is simulated at two nominal energy configurations of 6 MV and 10 MV for varying number of histories from 10^6^ to more than 10^8^. The dose is tallied on a homogeneous water phantom with dimensions 16.2 × 16.2 × 31.0 cm^3^ at a source-to-surface-distance of 100.0 cm. For each nominal energy setting, two initial electron beam energies are configured to reproduce the measured percent depth dose (PDD) distribution. Once the initial beam energy is fixed, several beam configurations are sequentially simulated to determine the parameters yielding good agreement with the measured lateral dose profiles. The simulated dose profiles are compared with the Varian Golden Beam Data Set (GBDS) using the gamma-index analysis method incorporating the dose-difference and distance-to-agreement criteria. The simulations are run on Pentium-type computers while the tuned 10 MV beam configuration is simulated at more than 10^8^ histories using a virtual server in the Amazon.com Elastic Compute Cloud.

**Results:**

The initial electron beam energy configuration that will likely reproduce the measured PDD is determined by comparing directly the gamma-index analysis results of two different beam configurations. The configuration is indicated to yield good agreement with data if the gamma-index passing rates using the 1%/1 mm criteria generally increase as the number of histories is increased. Additionally at the highest number of histories, the matching configuration gives a much higher passing rate at the 1%/1 mm acceptance criteria over the other competing configuration. With the matching initial electron beam energy known, this input to the subsequent simulations allows the fine-tuning of the lateral beam profiles to proceed at a fixed yet lower number of histories. In a three-stage serial optimization procedure, the first remaining beam parameter is varied and the highest passing rate at the 1%/1 mm criteria is determined. This optimum value is input to the second stage and the procedure is repeated until all the remaining beam parameters are optimized. The final tuned beam configuration is then simulated at much higher number of histories and the good agreement with the measured dose distributions is verified.

**Conclusions:**

As physical nature is not stingy, it reveals at low statistics what is hidden at high statistics. In the matter of fine-tuning a linac to conform with measurements, this characteristic is exploited directly by the PRIMO software package. PRIMO is an automated, self-contained and full Monte Carlo linac simulator and dose calculator. It embeds the gamma-index analysis toolkit which can be used to determine all the parameters of the initial electron beam configuration at relatively lower number of histories before the full simulation is run at very high statistics. In running the full simulation, the Amazon.com compute cloud proves to be a very cost-effective and reliable platform. These results are significant because of the time required to run full-blown simulations especially for resource-deficient communities where there could just be one computer as their sole workhorse.

## Introduction

In external photon and electron beam radiotherapy, the Monte Carlo (MC) method of radiation transport is generally considered to provide the most accurate estimate of the dose distribution. However the method is beset with two serious drawbacks. The first is the long computation time which renders it impractical for clinical usage and the second is the significant amount of work and expertise required to set up a MC simulation from scratch. Based on the general purpose MC code PENELOPE 2011, the self-contained, freely-distributed windows-based software package called PRIMO is designed to eliminate these hindrances [1–4].

As an automated and complete linac simulator and dose calculator for a variety of Varian and Elekta linacs, one of PRIMO’s outstanding features is its intuitive graphical user interface that enables a new user to seamlessly configure and execute the linac simulation. Prior knowledge of the physics of Monte Carlo is not a requirement although advanced users are given access to modify all the configuration files governing the linac simulation. Moreover the user does not input any geometrical information into the program since the linac geometries are already included in the package. The list of PRIMO’s other desirable features and benefits is long: option to output phase-space files (PSF), ability to import compliant external PSFs, a menu of variance-reduction techniques to reduce simulation time and the ability to distribute the simulations among the cores in a single computer among other capabilities.

Once the simulation is completed, the numerical tools within the interface can be accessed to analyze the output PSFs and the absorbed dose tallied in a binned water phantom or in a patient’s computerized tomography. Also accessible within the graphical user interface is the gamma-index analysis toolkit which enables the comparison of the simulated percent depth dose and lateral dose profiles with experimental values. The simulated 3D dose profiles must match with the measurements within the accepted limits of dosimetric uncertainty. When the matching succeeds and the experimental data are reproduced, the primary beam parameters of a linac have therefore been tuned for a given nominal energy. With this identified beam configuration, longer simulation with huge number of histories can then be run where a library of phase-space files of the upper part of the linac is generated. This library can be re-used in subsequent simulations of the linac with a substantially reduced simulation time.

Since the PRIMO code does not provide a beam configuration algorithm, the user must perform several simulations, each time varying the initial beam parameters, in order to reproduce the experimental dose profiles. This is a lengthy, time-consuming and resource-intensive process and is therefore a significant consideration for those with limited computational resources.

The absence of a beam configuration algorithm yet provides an instructive exercise for users in fine-tuning the linac beam. For example, our meager computational hardware does not allow unlimited number of simulations and for extended periods. Porting the linac simulations to the cloud is one strategy adopted in order to extend and augment our computing capabilities.

In this paper it is shown that the linac beam fine-tuning process can be truncated by using the gamma-index analysis results in the comparison of simulated and experimental dose profiles. A truncated process in fine-tuning the linac beam results to a reduction in computing time and this is important to attain compatibility with computing capabilities especially for resource-deficient communities.

## Materials and methods

The linac simulation in PRIMO is intuitively separated by geometrical segments. The photon beam generated from the linac head proceeds in three stages: starting from the electron beam source upstream of movable collimators (s1) then through the collimators themselves and all components of the lower part of the linac (s2). Then finally the dose distribution is computed in a binned water phantom located downstream (s3). In segment s2, the splitting-roulette variance-reduction option is chosen and fitted to the chosen field size while in segment s3, simple splitting in the phantom is also enabled with the splitting factor set to the value 20. In this study, the simulations of segments s1,s2 and s3 are done in succession. PSFs are output for simulations involving larger number of histories.

Using PRIMO version 0.1.5.1307, a Varian Clinac 2100 is simulated for varying number of histories at two nominal energy settings of 6 MV and 10 MV. Using one field of size 10 × 10 cm^2^, the dose is tallied on a homogeneous water phantom with dimensions 16.2 × 16.2 × 31.0 cm^3^ at a source-to surface distance of 100.0 cm. The dose-scoring bin size was set to 0.2 × 0.2 × 0.2 cm^3^.

For each nominal energy setting, two initial electron beam energies are configured: the first at the default value given by the PRIMO software and a second one at a higher electron beam energy. For the 6 MV nominal energy, the initial electron beam energy is configured at 5.40 MeV and 6.26 MeV while for the 10 MV, it is configured at 10.5 MeV and 10.7 MeV. In tuning the initial electron beam energy, the other beam parameters such as the full-width-half-maximum (FWHM) of the primary energy distribution, the focal spot FWHM and the beam divergence are each set to default values of zero.

In order to determine which initial electron beam energy will reproduce the measured PDD at a given nominal energy, the gamma-index passing rates are compared for simulations carried out at varying number of histories from 2 × 10^6^ to more than 6.5 × 10^7^ for 10 MV and up to 10^8^ histories for 6 MV. If the gamma-index passing rates at the 1%/1 mm criteria generally increase as the number of histories are increased, then this is the initial electron beam energy configuration that will likely reproduce the measurements.

With the optimal initial electron beam energy as input to further simulations, the fine-tuning of the three remaining electron beam parameters to reproduce the measured lateral dose profile proceeds through three serial stages of simulations. At each stage the simulations are carried out at a constant 2.5 × 10^6^ number of histories.

In the first stage only the energy FWHM is varied while the rest of the beam parameters are kept at default values of zero. The optimized configuration is that which gives the highest gamma-index passing rate using the 1%/1 mm criteria and is input to the next stage. In the second stage, with the optimized initial electron beam energy and energy FWHM as inputs while keeping the last parameter at zero default value, the focal spot FWHM is varied until the gamma-index passing rate reaches a maximum. Then finally, the beam divergence is varied to determine the configuration that gives the highest gamma-index passing rate. The final stage then provides the tuned beam configuration which is simulated to much higher number of histories.

The simulations are run using a student desktop computer (12-core 12 GB memory) and a laptop computer (4-core 16 GB memory) with × 86 Pentium-type processors. In the entire procedure of tuning the initial electron beam energy however, one and only one computing machine is dedicated for a particular nominal energy. For tuning the initial electron beam of the 6 MV configuration, the data of which are shown in Tables 1, 2, 3 and 4, only the desktop computer is used. The laptop computer is used for tuning the 10 MV configuration, the data of which are given in Tables 8, 9, 10 and 11. For the fine-tuning of the lateral dose profiles, only the desktop computer is used for both the 6 MV and 10 MV nominal configurations.

**Table 1:** Percentage of PDD dose points passing the three Γ-criteria. for varying histories at an initial beam energy of 6.26 MeV

Number of Histories (10^6^)	Average Dose Uncertainty (%)	3 % /3mm (%)	2 % /2mm (%)	1 % /1mm (%)
2	36.4	91.2	57.5	17.5
5	18.0	97.4	74.0	24.7
10	12.1	100.0	88.0	38.0
25	7.9	100.0	99.7	74.7
100	4.1	100.0	100.0	84.1

**Table 2:** Percentage of PDD dose points passing the three Γ-criteria. for varying histories at an initial beam energy of 5.40 MeV

Number of Histories (10^6^)	Average Dose Uncertainty (%)	3 % /3mm (%)	2 % /2mm (%)	1 % /1mm (%)
2	50.2	100.0	91.2	47.1
5	27.3	90.6	56.2	19.2
10	15.5	75.0	31.8	11.4
25	9.6	90.3	54.2	14.6
100	5.1	99.4	72.4	18.5

**Table 3:** Percentage of lateral dose profile points passing the three Γ-criteria for varying histories at an initial beam energy of 6.26 MeV

Number of Histories (10^6^)	Average Dose Uncertainty (%)	3 % /3mm (%)	2 % /2mm (%)	1 % /1mm (%)
2	36.4	100.0	96.9	59.6
5	18.0	100.0	93.2	54.7
10	12.1	100.0	96.9	58.4
25	7.9	99.4	81.4	47.8
100	4.1	99.4	95.7	75.2

**Table 4:** Percentage of lateral dose profile points passing the three Γ-criteria for varying histories at an initial beam energy of 5.40 MeV

Number of Histories (10^6^)	Average Dose Uncertainty (%)	3 % /3mm (%)	2 % /2mm (%)	1 % /1mm (%)
2	50.2	95.0	66.5	38.5
5	27.3	98.8	70.8	42.9
10	15.5	100.0	98.8	64.6
25	9.6	98.8	79.5	42.9
100	5.1	100.0	98.1	77.6

The tuned beam configuration at 6 MV which is comprised by the four optimized beam parameters is simulated to more than 2.8 × 10^8^ histories using the desktop computer. It took about 9.6 × 10^5^ s or more than 11 days to simulate segment s1 alone and another 16 h to complete the simulations of the s2 and s3 segments. The splitting factor at s3 segment is set to the value of 100 to keep the level of dose uncertainty under 1.5%.

Due to power interruption in our campus, many of our simulation attempts were aborted. Power outages, both scheduled and unscheduled, are a common occurrence in our locality. Porting some of the simulations to the Amazon.com cloud has helped mitigate this problem [5].

### Running PRIMO in the Amazon.com elastic compute cloud

Without investing in expensive hardware upfront, windows virtual machines can be launched, configured and connected using the Amazon Elastic Compute Cloud (EC2) where PRIMO simulations can be deployed in mere minutes.

Amazon EC2 is the central core of Amazon.com’s on-demand cloud-computing platform. It provides scalable computing capacity where one can launch as many or as few virtual servers as one needs, configure security and networking, and manage storage through a web-based user interface. The EC2 virtual computers also called instances, come with preconfigured templates known as Amazon Machine Images (AMI) which contain the operating system and other software [6]. This allows a user to install and run specific applications such as PRIMO in just a few minutes via a client computer using the remote desktop protocol. One key difference however between a real server and an Amazon EC2 server is that when an instance is terminated, the virtual server and its data are no longer available.

Access to Amazon EC2 is on a subscription basis and one is charged for the usage on a per-hour basis. There is a free-tier option for new account holders for the first twelve months of use where there are no charges incurred. The free-tier option allows a new user to run a micro-sized (1 CPU, 1 GB memory) server in the cloud, with storage and bandwidth completely free of charges for one year, provided the monthly usage does not exceed 720 h.

The PRIMO program of the same version is first installed and run in a Windows 2016 base server AMI with instance type in the free-tier option. The simulation of a 6 MV Varian C2100 linac with 6.26 MeV initial energy is configured to run for 720 h. After a full 30 days of non-stop calculations, the s1 segment is completed with a total of more than 1.07 × 10^7^ histories.

In order to simulate huge number of histories in a much shorter period, a faster EC2 instance is launched – the C5 instance, introduced in 2017, powered by 3.0 GHz Intel Xeon scalable processors. With this type of architecture, a Windows 2019 base server AMI with 32 cores and 64 GB memory takes more than 53 h to complete the s1 segment of more than 2.75 × 10^8^ histories and another 7 h to finish the s2 and s3 segments in the binned water phantom for the tuned beam 10 MV configuration.

The simulations in the Amazon cloud including the bandwidth for file transfers to the local computer are totally free of any charges because of the free-tier option and other educational credits offered by Amazon Web Services (AWS) [7]. Remarkable as that may seem, what is really phenomenal is the fact that even without these educational credits, the charges for the full simulation of the tuned beam 10 MV configuration amount to just around two hundred dollars (US$200) at 2019 prices. Nevertheless since the educational credits available are not unlimited, the use of AWS EC2 platform may no longer be recommended for the fine-tuning of the beam profiles since the entire procedure involves many stages; unless of course the financial resources are not an issue.

### Gamma-index analysis method

The simulated 3D dose distributions are compared to a given experimental result consisting of the lateral dose profile and percent depth dose curves measured on a Varian Clinac2100. The gamma-index analysis method incorporating the dose-difference and the distance-to-agreement criteria is used in the comparison. For a given experimental point *p* and the dose at that point *d*_*e*_*(p),* the gamma-index, Γ, is evaluated as

$$ \Gamma =\min \left\{\sqrt{{\left(\frac{\Delta  {d}_i}{\Delta  D}\right)}^2+{\left(\frac{\Delta  {s}_i}{\Delta  S}\right)}^2}\right\}, $$

where the arbitrary constants *∆D* and *∆S* are known as the acceptance criteria for the dose-difference and for the distance-to-agreement, respectively. The term *∆d*_*i*_ is the difference between the measured dose at that point *d*_*e*_*(p)* and the simulated dose at a certain point *p*_*i*_. The term *∆s*_*i*_ is the distance between *p* and *p*_*i*_. The minimum of the expression in curly braces is evaluated for the set of points {*p*_*i*_} where the set contains the points in the vicinity of *p* that extends up to a distance of 5*∆S* or a maximum of 1.5 cm. The resolution in each spatial direction is enhanced to one-fifth of the bin size by tri-linear interpolation of the simulated dose distribution [8–10].

If the gamma-index, Γ, is equal to or less than 1, the calculation is said to pass the gamma analysis test using a chosen acceptance criterion. On the other hand, if Γ is greater than 1, it has failed the test.

The experimental results are taken from the Varian GBDS which contains basic beam data input files such as depth dose scans, profile scans and output factors for a given modality and nominal energy. The data set is valid for Varian Clinac21/23EX Series medical linear accelerators and constitutes the minimum required beam data for configuring the treatment planning system for dose calculations [11].

## Results

### 6 MV nominal energy

Two initial electron beam energies are configured to fine-tune the Varian Clinac2100 at 6 MV. Table 1 shows the percentage of PDD dose points passing the three gamma-index analysis acceptance criteria for varying histories at an initial electron beam energy of 6.26 MeV. The passing rates systematically increase using all three criteria as the number of histories increases. For 10^8^ histories, the passing rate at the 1%/1 mm criteria is more than 84% giving a good agreement of the measured and simulated PDD data.

For the default initial electron beam energy configuration at 5.40 MeV, Table 2 shows the gamma-index passing rates using three different acceptance criteria in the comparison of the measured and simulated PDD for varying number of histories. From 2.0 × 10^6^ up to 10^7^ histories, the passing rates decrease in all three criteria. The passing rates manage to increase in all the acceptance criteria as the number of histories increases from 2.5 × 10^7^ up to 10^8^. The rate of increase however is quite slow. For 10^8^ histories, the gamma-index analysis gives a passing rate of just over 18% at the 1%/1 mm criteria, revealing a wide mismatch between the measured and simulated PDD data.

Linear regression analysis can also be applied to compare the data of Tables 1 and 2. For Table 1, the Pearson correlation coefficient, *r*, between the average statistical uncertainty and the gamma-index passing rate at 1%/1 mm criteria is equal to −0.84. This value denotes a strong negative correlation between the two quantities. In comparison, Table 2 gives a value *r* = + 0.88 which is an equally strong correlation but in the opposite direction.

Comparing the data of Tables 1 and 2, the configuration at the higher initial electron beam energy of 6.26 MeV rather than at the default setting of 5.40 MeV is considered to most likely reproduce the measured PDD at much higher statistics. The value of 6.26 MeV for the initial electron beam energy is then used for the fine-tuning of the lateral dose profiles.

The results of the gamma-index analysis for the comparison of the measured and lateral dose profiles at the two initial electron beam energies are shown in Table 3 and Table 4. Given the level of dose uncertainty, there is no significant difference in the passing rates of the lateral dose distribution between the two initial electron beam energy configurations. The passing rates also do not show a systematic increase or decrease with increasing number of histories at both configurations.

With the initial electron beam energy now fixed at 6.26 MeV, the three remaining electron beam parameters are optimized in three serial stages in order to reproduce the measured lateral dose profiles. Several beam configurations are chosen and simulated for 2.5 × 10^7^ histories at each beam configuration. At 6 MV, the Varian GBDS gives one profile scan measurement for the 10 × 10 cm^2^ field at each of these depths: 1.6 cm, 5.0 cm, 10.0 cm, 20.0 cm and 30.0 cm. The simulated lateral dose profiles are then compared with each and every scan depth measurement. The data shown in the subsequent tables are those which give the best comparison of the measured and simulated lateral beam profiles.

Table 5 shows the results of gamma-index analysis in which the passing rate at the 1%/1 mm criteria is highest for each varying energy FWHM configuration with the initial electron beam energy fixed at 6.26 MeV, the focal spot FWHM and beam divergence are at default values of 0. The passing rate peaks at 70.8% when the energy FWHM value is 0.150 MeV. This value is then input to the second stage of the simulation where the focal spot size is varied while the beam divergence is kept at the default value of 0.

**Table 5:** Percentage of lateral dose profile points passing the three Γ-criteria with the initial electron beam energy fixed at 6.26 MeV, the focal spot FWHM and beam divergence are at default values of 0.The number of histories is 2.5 × 10^7^

Energy FWHM (MeV)	Average Dose Uncertainty (%)	3 % /3mm (%)	2 % /2mm (%)	1 % /1mm (%)
0.120	7.9	100.0	92.6	53.4
0.140	7.9	100.0	99.4	68.3
0.150	7.9	100.0	99.4	70.8
0.160	7.9	100.0	98.1	65.8

In Table 6, the 1%/1 mm gamma-index passing rate peaks at 78.9% when the focal spot size is 0.15 cm. This value is then input to the third stage of the simulations where the beam divergence is varied. As shown in Table 7, the highest value of the 1%/1 mm passing rate which is 75.8% is found when the beam divergence is 3°. The last stage therefore provides the tuned beam configuration for the 6 MV nominal energy: initial electron beam energy = 6.26 MeV, energy FWHM = 0.150 MeV, focal spot FWHM = 0.15 cm and beam divergence = 3^0^. The final simulations at more than 2.8 × 10^8^ histories is then undertaken using the desktop computer.

**Table 6:** Percentage of lateral dose profile points passing the three Γ-criteria with the initial electron beam energy fixed at 6.26 MeV, the energy FWHM at 0.150 MeV and beam divergence at default value of 0.The number of histories is 2.5 × 10^7^

Focal Spot FWHM (cm)	Average Dose Uncertainty (%)	3 % /3mm (%)	2 % /2mm (%)	1 % /1mm (%)
0.10	7.9	100.0	98.8	67.7
0.12	7.9	100.0	100.0	68.9
0.14	7.9	100.0	99.4	72.1
0.15	7.9	100.0	100.0	78.9
0.16	7.9	100.0	93.8	75.2

**Table 7:** Percentage of lateral dose profile points passing the three Γ-criteria with the initial electron beam energy fixed at 6.26 MeV, the energy FWHM at 0.150 MeV, and the focal spot FWHM at 0.15 cm. The number of histories is 2.5 × 10^7^

Beam Divergence	Average Dose Uncertainty (%)	3 % /3mm (%)	2 % /2mm (%)	1 % /1mm (%)
1^0^	8.0	100.0	93.8	48.4
2^0^	8.0	100.0	87.6	56.5
3^0^	8.1	100.0	98.8	75.8
4^0^	8.2	100.0	98.8	60.2

**Fig. 1:**
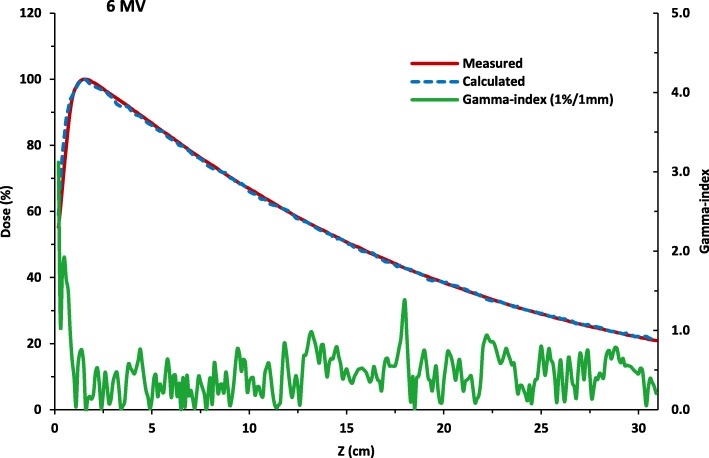
The calculated percent depth dose curve compared with measurements and its associated gamma-index (1 % /1mm) as a function of depth . The simulation is run for more than 2.8 × 10^8^ number of histories at the beam configuration of 6.26 MeV initial electron beam energy, energy FWHM of 0.150 MeV, 0.15 cm focal spot FWHM and 3^0^ beam divergence. At the 1 % /1mm acceptance criteria, the passing rate is 97.1% . The average statistical uncertainty in the dose is 1.4%

**Fig. 2:**
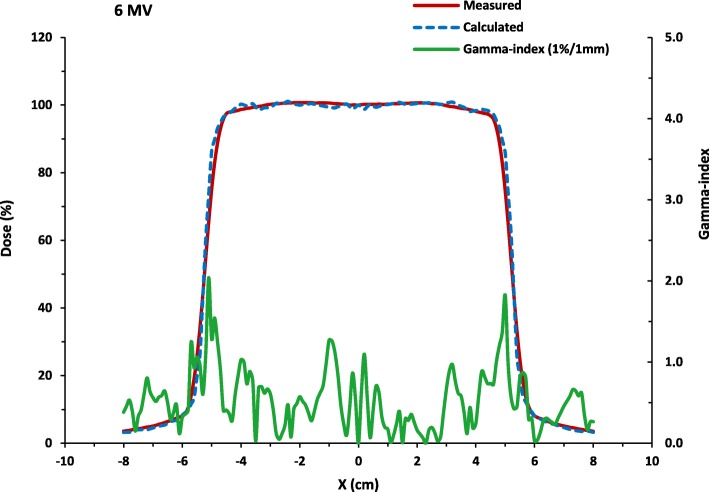
The calculated lateral dose profile compared with measurements and its associated gamma-index (1 % /1mm) as a function of depth . The simulation is run for more than 2.8 × 10^8^ number of histories at the beam configuration: 6.26 MeV initial electron beam energy, 0.150 MeV energy FWHM, 0.15 cm focal spot FWHM and 3^0^ beam divergence. The gamma-index passing rate using the 1 % /1mm criteria is 89.4% while the average statistical uncertainty in the dose is 1.4%

The results for the final simulations of the tuned beam configuration at 6MV are shown in Fig. 1 for the comparison of the measured and simulated PDD data and Fig. 2 for the comparison of the lateral dose profile. The plots show graphically the good agreement with measurements and simulated data. The gamma-index analysis passing rates for the PDD distribution is 97.1% while for the lateral dose profile, it is more than 89.4% using the 1%/1 mm criteria. The average statistical uncertainty in the dose is 1.4%.

### 10 MV Nominal Energy

Two initial electron beam energies are also configured to tune the Varian Clinac2100 at 10 MV: 10.7 MeV and 10.5 MeV which is the default value given by PRIMO. Table 8 shows the percentage of PDD dose points passing the three gamma-index analysis acceptance criteria for varying histories at an initial electron beam energy of 10.7 MeV. The passing rates using all three criteria increase as the number of histories increase from 5 × 10^6^ to 10^7^ histories but steadily declines as the number of histories is increased to more than 6.5 × 10^7^.

**Table 8:** Percentage of PDD dose points passing the three Γ-criteria for varying histories at an initial beam energy of 10.7 MeV

Number of Histories (10^6^)	Average Dose Uncertainty (%)	3 % /3mm (%)	2 % /2mm (%)	1 % /1mm (%)
5	12.4	99.0	86.1	42.9
10	9.1	100.0	98.7	65.2
25	5.9	100.0	97.7	60.3
65.06	3.7	100.0	93.6	51.6

In Table 9, the gamma-index analysis passing rates are shown where the initial electron beam energy is configured at the default value of 10.5 MeV. Except for the dip of 0.3% at 2%/2 mm criteria at the highest number of histories, the gamma-index passing rates generally increase as the number of histories is increased. Since at a level in excess of 6.5 × 10^7^ number of histories, the passing rate using 1%/1 mm criteria is more than 84%, the configuration at 10.5 MeV initial electron beam energy is indicated to reproduce the measured PDD curve rather than at the higher energy of 10.7 MeV.

**Table 9:** Percentage of PDD dose points passing the three Γ-criteria for varying histories at an initial beam energy of 10.5 MeV

Number of Histories (10^6^)	Average Dose Uncertainty (%)	3 % /3mm (%)	2 % /2mm (%)	1 % /1mm (%)
5	12.8	99.4	73.6	29.0
10	9.4	99.4	75.2	30.0
25	6.1	100.0	100.0	77.1
65.06	3.8	100.0	99.7	84.2

The above conclusion is verified when a linear regression analysis is applied to compare the data of Tables 8 and 9. For the data at an initial electron beam energy of 10.7 MeV as shown in Table 8, the Pearson correlation coefficient, *r*, between the average statistical uncertainty and the gamma-index passing rate at 1%/1 mm criteria is equal to - 0.34. This value denotes a weak negative correlation between the two quantities. On the other hand, Table 9 gives a value of *r* = − 0.93 which shows a strong negative correlation between the two quantities.

At the two respective initial electron beam energies, the gamma-index analysis passing rates for the lateral dose profiles are shown in Table 10 and Table 11. Similar to the case of the 6 MV nominal energy, the lateral dose profiles for the two different beam configurations do not show significant difference given the level of the dose uncertainty. Nor do the passing rates show a dependence on the number of histories at both initial beam energy configurations.

**Table 10:** Percentage of lateral dose points passing the three Γ-criteria for varying histories at an initial beam energy of 10.7 MeV

Number of Histories (10^6^)	Average Dose Uncertainty (%)	3 % /3mm (%)	2 % /2mm (%)	1 % /1mm (%)
5	12.4	99.4	85.1	62.1
10	9.1	98.8	85.1	54.0
25	5.9	100.0	100.0	74.5
65.06	3.7	100.0	90.7	55.9

**Table 11:** Percentage of lateral dose points passing the three Γ-criteria for varying histories at an initial beam energy of 10.5 MeV

Number of Histories (10^6^)	Average Dose Uncertainty (%)	3 % /3mm (%)	2 % /2mm (%)	1 % /1mm (%)
5	12.8	96.9	93.8	67.7
10	9.4	100.0	93.8	67.7
25	6.1	100.0	100.0	78.9
65.06	3.8	99.9	87.0	62.1

The lateral dose profiles at the 10 MV nominal energy are then used to fine-tune the other three remaining beam parameters using the same three-stage optimization procedure described in Section 2 and earlier applied to the case of the 6 MV nominal energy configuration. With the initial electron beam energy fixed at 10.5 MeV, several beam configurations are simulated at 2.5 × 10^7^ histories. The simulated lateral dose profiles are compared with the Varian GBDS which at 10 MV and 10 × 10 cm^2^ field, gives one measurement at each of five scan depths: 2.4 cm, 5.0 cm, 10.0 cm, 20.0 cm and 30.0 cm. The values given in the subsequent tables are the gamma-index analysis passing rates using the 1%1/mm criteria at the scan depth where the comparison with data gives the best value.

As shown in Table 12, the passing rate at 1%/1 mm criteria is highest at energy FWHM = 0.140 MeV. With this value fixed, the optimization of the last two remaining beam parameters then proceeds sequentially in two stages. The highest passing rate at 1%/1 mm criteria is achieved for the focal spot FWHM value of 0.12 cm. At the last step with the optimized values of initial beam energy, energy FWHM and focal spot size as inputs, the highest passing rate of 78.9% at 1%/1 mm criteria is obtained when the beam divergence is equal to 1^0^. The data tables for these steps are omitted here for brevity.

**Table 12:** Percentage of lateral dose profile points passing the three Γ-criteria with the initial electron beam energy fixed at 10.5 MeV, the focal spot FWHM and beam divergence are at default values of 0.The number of histories is 2.5 × 10^7^

Energy FWHM (MeV)	Average Dose Uncertainty (%)	3 % /3mm (%)	2 % /2mm (%)	1 % /1mm (%)
0.100	5.9	100.0	96.9	72.1
0.120	5.9	100.0	98.8	78.3
0.140	6.0	100.0	98.8	80.1
0.150	5.9	100.0	98.1	75.2

Table 13 below however shows that the energy FWHM configuration with 0.120 MeV, compared with 0.140 MeV, is the better configuration since the passing rate at 1%/1 mm criteria is highest in four scan depths out of a total five. Additionally, comparing the gamma-index passing rate for the optimized tuned-beam configurations, it is higher at 0.120 MeV (81.4%) than that for 0.140 MeV (78.9%).

**Table 13:** Comparison of the percentage of lateral dose profile points passing the 1%/1 mm criteria for the two energy FWHM configurations at 0.120 MeV and 0.140 MeV at various scan depths. The initial electron beam energy is fixed at 10.5 MeV, the focal spot FWHM and beam divergence are at default values of 0. The number of histories is 2.5 × 10^7^

Energy FWHM (MeV)	Average Dose Uncertainty (%)	Passing rates at 1%/1 mm criteria for various depths (%)				
		2.4 cm	5.0 cm	10.0 cm	20.0 cm	30.0 cm
0.120	5.9	59.0	74.5	39.8	78.3	45.3
0.140	5.9	80.1	36.6	14.3	36.0	9.9

Fixing the energy FWHM at 0.120 MeV, the last two remaining beam parameters are then sequentially optimized in two stages. As shown in Tables 14 and 15, the highest passing rate of 81.4% at 1%/1 mm criteria is achieved at this final configuration for the 10.5 MeV initial beam energy: energy FWHM = 0.120 MeV, focal spot FWHM = 0.12 cm and beam divergence = 1^0^.

**Table 14:** Percentage of lateral dose profile points passing the three Γ-criteria with the initial electron beam energy fixed at 10.5 MeV, the energy FWHM at 0.120 MeV and beam divergence at default value of 0.The number of histories is 2.5 × 10^7^

Focal Spot FWHM (cm)	Average Dose Uncertainty (%)	3 % /3mm (%)	2 % /2mm (%)	1 % /1mm (%)
0.10	5.9	100.0	97.5	71.4
0.12	5.9	100.0	100.0	73.9
0.13	5.9	99.4	91.3	66.5

**Table 15:** Percentage of lateral dose profile points passing the three Γ-criteria with the initial electron beam energy fixed at 10.5 MeV, the energy FWHM at 0.120 MeV, and the focal spot FWHM at 0.12 cm. The number of histories is 2.5 × 10^7^

Beam Divergence	Average Dose Uncertainty (%)	3 % /3mm (%)	2 % /2mm (%)	1 % /1mm (%)
1^0^	6.0	100.0	100.0	81.4
2^0^	6.0	100.0	95.0	74.5

Having found this optimized beam configuration at 10MV nominal energy, further simulations at very high statistics is then carried out with confidence. With more than 2.75 × 10^8^ histories, the tuned beam configuration is simulated using a virtual server in the Amazon.com compute cloud.

**Fig. 3:**
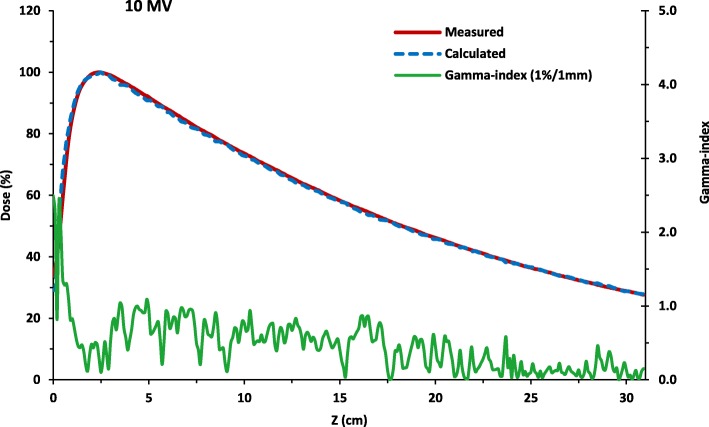
The calculated percent depth dose curve compared with measurements and its associated gamma-index (1 % /1mm) as a function of depth . The simulation is run for more than 2.75 × 10^8^ number of histories at the beam configuration: 10.5 MeV initial electron beam energy, 0.120 MeV energy FWHM, 0.12 cm focal spot FWHM and 1^0^ beam divergence. The gamma-index passing rate using the 1 % /1mm criteria is 96.8% while the average statistical uncertainty in the dose is 1.0%

**Fig. 4:**
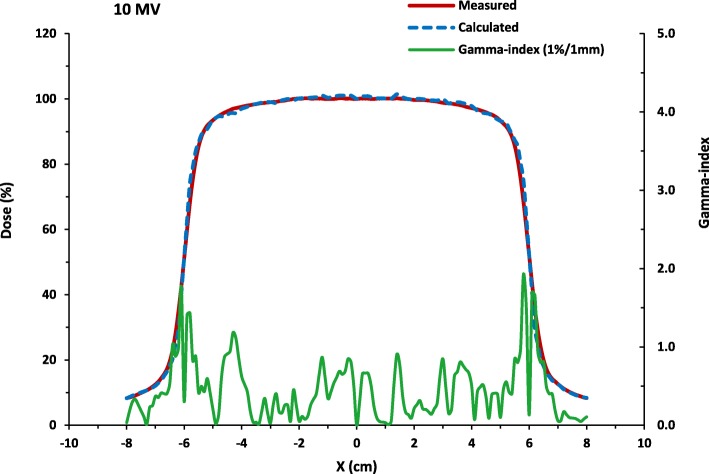
The calculated lateral dose profile compared with measurements and its associated gamma-index (1 % /1mm) as a function of depth . The simulation is run for more than 2.75 × 10^8^ number of histories at the beam configuration: 10.5 MeV initial electron beam energy, 0.120 MeV energy FWHM, 0.12 cm focal spot FWHM and 1^0^ beam divergence. The gamma-index passing rate using the 1 % /1mm criteria is 93.8% while the average statistical uncertainty in the dose is 1.0%

Fig. 3 and Fig. 4 show the results of the simulation where the good agreement of the simulated profiles with the Varian GBDS measurements is verified. The gamma-index passing rate using the 1 % /1mm criteria is 96.8% for the PDD distribution and 93.8% for the lateral dose profile. The average statistical uncertainty in the dose is 1.0%.

## Discussions

The dose distribution resulting from a Monte Carlo simulation is primarily a function of the initial linac beam configuration consisting of the parameters such as the initial electron beam energy, energy width, the focal spot size of the beam and its divergence. Although the PRIMO code does not provide a beam configuration algorithm, it gives default values for these parameters. The user thus needs to perform several simulations by changing these default parameters to obtain a good match between the simulated and measured dose distributions.

In this study, the standard procedure for fine-tuning a linac photon beam is adopted. The first step is to determine the primary electron beam energy that reproduces the experimental percent depth dose curve. Once this optimal energy setting is found, this energy configuration is input in the succeeding simulations when varying the other remaining beam parameters until the experimental lateral dose profile is also sufficiently reproduced.

In the standard procedure however, much reliance is placed on the visual or graphical comparison of the simulated with the measured 3D dose profiles. The gamma-index analysis method complements the standard procedure thereby eliminating a great portion of it which involves trial-and-error.

In the gamma-index analysis method, simulations of two competing initial electron beam energy configurations are run at increasing number of histories and their gamma-index passing rates at 1%/1 mm criteria are directly compared. If increasing number of histories which corresponds to low average statistical uncertainties, will result to higher gamma-index passing rates, this will indicate a closer match with the experimental PDD distribution.

Since finding the matching initial electron beam energy is of paramount importance, it is imperative to run simulations at sufficiently high number of histories. In practice this means undertaking simulations for up to 10^8^ number of histories in the case of 6 MV corresponding to about 5% average statistical uncertainties in the dose. At this level the highest passing rate in the 1%/1 mm criteria will be more than 80% and the Pearson correlation coefficient, *r*, between the average statistical uncertainty and the 1%/1 mm passing rate will yield a value better than *r* = − 0.80. That is to say that the strong negative correlation between the two quantities may be attributed to a better initial electron beam energy configuration compared to the competing configuration which gives either a weak negative correlation or a positive correlation for the two quantities.

Having thus found the optimal setting of the primary electron beam energy, the simulations to fine-tune the lateral beam profiles can be undertaken at a fixed yet much lower number of histories of 2.5 × 10^6^. At this number, the average statistical uncertainty in the dose is still about 6% and 7.9% for the case of the 10 MV and 6 MV configurations respectively. On a practical note, the time required to run the s1 segment alone using the faster desktop computer is around 24 h which makes the procedure still feasible even given the lengthy three-stage serial process in fine-tuning the lateral beam profiles.

As for the tuned beam configurations at both nominal energy settings, it maybe remarked that the gamma-index passing rates for the lateral beam profiles are generally lower than that for the PDD distribution. In order to attain 1%/1 mm gamma-index passing rates at the level of 95% for the lateral beam profiles, quite huge number of histories are required for the simulations. High dose gradients characterize the penumbrae of the lateral beam profiles while a region of this kind exists only at the start of the dose buildup region for the PDD curve. The regions of high dose gradients pull down the passing rates to lower values. Simulations of the order of 10^9^ number of histories will likely even out this effect but unfortunately such is beyond the scope of our computing resources.

Although the results of this study cover new practical grounds in the conduct of fine-tuning a linac photon beam, admittedly much more remains to be desired. The basic assumption that the gamma-index passing rates at the1%/1 mm criteria generally increase as the number of histories are increased if the initial electron beam configuration is a suitable match has to be tested for varying field sizes both bigger and smaller than the 10 × 10 cm^2^ field considered here. Moreover the reliability of the assumption has to investigated and analyzed for the other initial beam parameters such as the energy width, focal spot and beam divergence. Once these measures are undertaken and completed, the apparent role of the level of uncertainties and the Pearson correlation coefficient vis-à-vis the gamma-index fine-tuning method may be plainly delineated. Nonetheless these recommendations demand a computing capability beyond our current resources.

Evidently another linac type other than the Varian machine studied here has to be utilized in order to probe further the validity of the basic assumption. For this reason, a study for the fine-tuning of an Elekta linac is being undertaken at the same nominal photon energies expecting to arrive at a machine-independent conclusion.

Finally, attaining a value of the 1%/1 mm gamma-index passing rates beyond 95% for the PDD distribution may be necessary for studies such as small field size output factors and volume effect of detectors which are dependent on point dose measurements.

The gamma-index analysis toolkit embedded inside the PRIMO software package leads to a systematic procedure to determine effectively at lower statistics the full beam configuration which will sufficiently reproduce the measured dose distributions at very high number of histories. This has therefore resulted to a relatively shorter linac photon beam fine-tuning process.

## Conclusion

PRIMO is an automated, self-contained and full Monte Carlo linac simulator and dose calculator and exceptionally user-friendly. The first step in using Monte Carlo simulations for clinical applications is fine-tuning the linac beam so as to match the simulated with measured dose profiles. This is a lengthy, time-consuming and resource-intensive process. Embedded in PRIMO are excellent features and tools which can truncate the linac beam tuning process to reduce simulation time. Apart from the default beam parameter values for each linac type which PRIMO reasonably suggests, the gamma-index analysis toolkit accessible within PRIMO’s graphical user interface provides detailed comparison between the calculated and measured dose profiles. The gamma-index analysis method can be exploited to effectively predict, at lower statistics, which beam configuration will likely reproduce the measured beam profiles. This is significant because of the time required to run simulations at huge numbers of histories and especially for resource-deficient communities, there are simply not enough computers to do the tasks. For such communities, porting full-blown simulations to the Amazon.com compute cloud provides a cost-effective and reliable platform.

## Data Availability

All source files, supporting data and materials that are not presented in the manuscript are available from the author on reasonable request.

## Abbreviations

AWS: Amazon Web Services
EC2: Elastic Compute Cloud
FWHM: Full-width-half-maximum
GBDS: Golden Beam Data Set
MC: Monte Carlo
NRCP: National Research Council of the Philippines
PDD: Percent depth dose
PSF: Phase-space File
